# Imaging-Based Screening of Deubiquitinating Proteases Identifies Otubain-1 as a Stabilizer of c-MYC

**DOI:** 10.3390/cancers14030806

**Published:** 2022-02-04

**Authors:** Shannon E. Moree, Laure Maneix, Polina Iakova, Fabio Stossi, Ergun Sahin, Andre Catic

**Affiliations:** 1 Department of Molecular and Cellular Biology, Baylor College of Medicine, Houston, TX 77030, USA; Shannon.Moree@bcm.edu (S.E.M.); Laure.Maneix@bcm.edu (L.M.); Polina.Iakova@bcm.edu (P.I.); stossi@bcm.edu (F.S.); 2 Huffington Center on Aging, Baylor College of Medicine, Houston, TX 77030, USA; esahin@bcm.edu; 3 Stem Cells and Regenerative Medicine Center, Baylor College of Medicine, Houston, TX 77030, USA; 4 Gulf Coast Consortia, Center for Advanced Microscopy and Image Informatics, Houston, TX 77030, USA; 5 Dan L. Duncan Comprehensive Cancer Center, Baylor College of Medicine, Houston, TX 77030, USA; 6 Center for Cell and Gene Therapy, Baylor College of Medicine, Houston, TX 77030, USA; 7 Michael E. DeBakey VA Medical Center, Houston, TX 77030, USA

**Keywords:** OTUB1, c-MYC, ubiquitin–proteasome system, deubiquitinating protease, genetic screen, high-content imaging, deubiquitinase, protein turnover, oncogene, protein degradation

## Abstract

**Simple Summary:**

The transcription factor c-MYC drives growth and proliferation in most cancers. The c-MYC protein is short-lived and rapidly degraded. However, deubiquitinating enzymes can counteract its degradation and increase the levels of this oncogenic protein. We altered the expression of 41 individual deubiquitinases and measured the abundance of c-MYC. We discovered that the deubiquitinating enzyme OTUB1 slows the degradation of the transcription factor c-MYC and increases its abundance in the nucleus. Further, patients with high expression of OTUB1 show poor clinical outcomes in the c-MYC-dependent cancer multiple myeloma. Finally, elevating OTUB1 expression in multiple myeloma cells stimulates more aggressive growth. Thus, inhibition of OTUB1 may blunt c-MYC activity, making it a promising target for cancer treatment.

**Abstract:**

The ubiquitin–proteasome pathway precisely controls the turnover of transcription factors in the nucleus, playing an important role in maintaining appropriate quantities of these regulatory proteins. The transcription factor c-MYC is essential for normal development and is a critical cancer driver. Despite being highly expressed in several tissues and malignancies, the c-MYC protein is also continuously targeted by the ubiquitin–proteasome pathway, which can either facilitate or inhibit c-MYC degradation. Deubiquitinating proteases can remove ubiquitin chains from target proteins and rescue them from proteasomal digestion. This study sought to determine novel elements of the ubiquitin–proteasome pathway that regulate c-MYC levels. We performed an overexpression screen with 41 human proteases to identify which deubiquitinases stabilize c-MYC. We discovered that the highly expressed Otubain-1 (OTUB1) protease increases c-MYC protein levels. Confirming its role in enhancing c-MYC activity, we found that elevated OTUB1 correlates with inferior clinical outcomes in the c-MYC-dependent cancer multiple myeloma, and overexpression of OTUB1 accelerates the growth of myeloma cells. In summary, our study identifies OTUB1 as a novel amplifier of the proto-oncogene c-MYC.

## 1. Introduction

Specificity is a key feature of protein biology. Proteins originate through translation of defined transcripts and are eliminated through precise removal by the ubiquitin–proteasome system. This pathway adds ubiquitin chains to mark proteins slated for degradation. The proteasome, a large protease complex, is responsible for the removal of most cytosolic and nuclear proteins [1]. The proteasome recognizes ubiquitin chains in which the C-termini are covalently bound to lysine 11 or 48 to identify elimination-prone proteins [2]. Ubiquitin is attached to target proteins through a multi-enzyme cascade. In the final step, ubiquitin ligases covalently transfer ubiquitin to the substrate poly-peptide. The mammalian genome encodes approximately 600–1000 ubiquitin ligases [3], which often form complexes to further increase the possible combinations of ubiquitin-transferring enzymes [4]. This variety ensures that ubiquitin specifically modifies the intended target proteins. While covalent, ubiquitination is reversible. Specific proteases, deubiquitinases, can edit or entirely remove ubiquitin chains from target proteins to alter their function or save them from degradation. The human genome may encode >100 deubiquitinating proteases, which are thought to display target specificity like their ubiquitin ligase counterparts [5,6]. Several deubiquitinases have been shown to be involved in cancer progression, including members of the ubiquitin carboxyl-terminal hydrolase (UCH) family, the ubiquitin specific protease (USP) family, and the Otubain protease (OTU) family [7,8,9].

Transcription factors are among the proteins most closely surveilled by the ubiquitin–proteasome system. Transcription factors have short half-lives, and their activity is often directly linked to their degradation, ensuring that the regulation of individual genes remains dynamic [10,11,12,13,14,15]. One of the best studied examples of a short-lived transcription factor whose activation also triggers its rapid degradation is c-MYC [16,17,18,19]. c-MYC controls a large fraction of genes, driving protein synthesis pathways, cell proliferation, and metabolic activation [20,21,22,23,24]. In many cancers, c-MYC acts as an oncogene, and elevated c-MYC levels contribute to transcriptional deregulation [25,26]. In healthy tissues, c-MYC is necessary for growth and healing but also accelerates aging [27].

Several ubiquitin ligases and deubiquitinases have been shown to act on c-MYC [28,29]. Cullin ligases represent the most diverse group of enzymes involved in ubiquitination. They form complexes with variable substrate receptors that are critical for recognition of specific target proteins. One such receptor is FBXW7 (or FBW7), which binds to c-MYC and triggers its ubiquitination, followed by proteasomal degradation. On the other hand, c-MYC-specific deubiquitinases can stabilize the c-MYC protein by rescuing it from degradation, making them attractive conceptual targets for the treatment of cancers. In fact, deubiquitinase inhibitors have been developed as therapy for cancer and other diseases [30,31]. Specifically, inhibiting these enzymes could decrease c-MYC levels and slow cancer growth.

Previous studies used gene depletion strategies to identify c-MYC-specific deubiquitinases [32,33,34]. While reducing the expression of these proteases diminished c-MYC levels, c-MYC levels are also reduced under generally cytotoxic conditions [35]. Therefore, non-specific growth inhibition by the depletion of a vital deubiquitinase could have similar effects and confound results using these approaches. Further, most of the previously detected deubiquitinases are expressed at low levels [28], calling into question a dominant role in controlling the highly abundant and dynamic c-MYC protein.

In this study, we performed an overexpression screen of 41 deubiquitinating proteases and assessed their impact on c-MYC protein levels. Our results identified the highly expressed Otubain-1 (OTUB1) as a novel deubiquitinase that stabilizes the c-MYC proto-oncogene.

## 2. Materials and Methods

### 2.1. Cloning of pLVX-Puro/ZsGreen1-c-MYC

pLVX-Puro (Cat. 632164, TakaraBio, San Jose, CA, USA) was digested using XbaI (Cat. R0145, New England Biolabs NEB, Ipswich, MA, USA), and empty ligation was minimized using Phosphatase (NEB, Cat. M0290). The open vector was then purified using the MinElute PCR Purification Kit (Cat. 28004, Qiagen, Germantown, MD, USA), blunted at room temperature using the Quick Blunting Kit from NEB (Cat. E1201), and heat inactivated for 10 min at 70 °C. The ZsGreen1 gene from pRetroX-IRES-ZsGreen1 (TakaraBio, Cat. 632520) and the c-MYC gene from a 293T cell-derived in-house cDNA library were cloned by PCR using Phusion High-Fidelity PCR Master Mix with HF Buffer (Cat. F531L, Thermo Scientific, Waltham, MA, USA). The PCR product was phosphorylated using T4 Polynucleotide Kinase (NEB, Cat. M0201). Blunt ligation was performed with the PCR-amplified and phosphorylated ZsGreen1-c-Myc fusion construct and XbaI-cut and blunted recipient vector using Quick Ligase (NEB, Cat. M2200S). The ligation mixture was transformed into Oneshot Stbl3 competent cells (Invitrogen, Cat. C7373-03) according to the recommended protocol and plated on LB + Ampicillin plates (100 μg/mL). The final construct was confirmed by 5′ sequencing at the BCM DNA Sequencing Core.

### 2.2. Cloning of the Deubiquitinase Library

The deubiquitinase (DUB) cDNA library was obtained from the Baylor College of Medicine Cell Based Assay Screening Service (CBASS) core. The cDNAs originated from the Ultimate ORF and human ORFeome collection v5.1 and v7.1. Libraries were provided in Gateway cloning vectors pENTR223 and pDONR, respectively. For this project, we modified the pRetroX-IRES-DsRedExpress vector (TakaraBio, Cat. 632521) to allow for the use of Gateway cloning to insert the cDNA proximal to the IRES site within the multiple cloning site. We used the Gateway Vector Conversion System with One Shot ccdB Survival 2 T1R Competent Cells kit from Invitrogen (Cat. 11828-029) to add a Gateway cassette containing the chloramphenicol resistance gene (CmR) and the ccdB gene flanked by attR1 and attR2 sites to our vector. The pRetroX-IRES-DsRedExpress vector was cut with BamHI-HF (NEB, Cat. R3136), PCR purified as above, blunted at room temperature, and heat inactivated for 10 min at 70 °C. Next, we treated it with rSAP (NEB, Cat. M0371) for 30 min at 37 °C and heat inactivated it at 65 °C. The product was run in a 1% agarose gel with SYBR Safe DNA gel stain and cut on an e-Gel station using blue light illumination. Gel pieces were purified using Zymoclean Gel DNA Recovery Kit (Cat. D4007, Zymo Research, Irvine, CA, USA). Purified DNA was ligated with Gateway C.1 from the Gateway Vector Conversion kit using the quick ligase kit. The ligation mixture was transformed into OneShot ccdB Survival competent cells according to protocol, plated onto LB + Amp and LB + Amp + 20 μg/mL Chloramphenicol, and incubated overnight. We used colony PCR to choose colonies based on size using primers designed and shown below to amplify from the vector in both directions. Positive colonies were sent for sequencing at the core.

pRetrox-Fwd: GGGGTTTTATGCGATGGAGT

pRetroX-Rev: AGGAACTGCTTCCTTCACGA

The DUB-cDNA-containing plasmids from the CBASS core were grown overnight in 50 μg/mL LB + Kanamycin for the Ultimate ORF library or LB + Spectinomycin for the ORFeome library and mini prepped using the QIAprep Spin Miniprep Kit (Qiagen, Cat. 27104). Each DUB was individually combined with the sequence-verified pRetroX-Gateway C.1-IRES-dsRed vector in Gateway cloning reactions using Gateway LR Clonase II Enzyme Mix (Invitrogen, Cat. 11791-020) according to the vendor’s protocol, transformed into Stbl3 competent cells, and plated on LB + Ampicillin plates for colony selection. Colony PCR was performed using Platinum HotStart PCR 2X Master Mix (Invitrogen, Cat. 1300-012) and sent for sequencing using the same primers as when cloning the Gateway C.1-containing vector. For the ORFeome clones, which did not contain STOP codons; vector-derived amino acids PTFLYKVVRSCPSPSPPPNVTGRSRLE- formed the C-termini of these sequences. 

### 2.3. Tissue Culture

HEK293T/17 (ATCC #CRL-11268, RRID: CVCL_1926) and HeLa (ATCC #CCL-2, RRID: CVCL_0030) cell lines were obtained from the Baylor College of Medicine Molecular and Cellular Biology Tissue Culture Laboratory and were initially purchased from the American Type Culture Collection (ATCC, Manassas, VA, USA) by the core. Cell lines were grown in sterile culture in Dulbecco’s Modified Eagle’s Medium (DMEM) (Cat. 10-017-CV, Corning, NY, USA) + 10% Fetal Bovine Serum (Cat. F0900-050, GenDepot, Katy, TX, USA) + 100 U/mL Penicillin/Streptomycin (Cat. 15140-122, Gibco, Waltham, MA, USA) in filtered lid cell culture flasks. MM.1S cells were cultured as previously published [36]. Cells were incubated at 37 °C, 5% CO_2_, with humidity, in tissue culture incubators that were regularly monitored for mycoplasma contamination. 

### 2.4. Generation of Transduced Cell Lines

For virus production encoding ZsGreen1-c-Myc, HEK293T cells were transfected in 6-well plates with a combination of the packaging construct pCMV-DeltaR8.9, a pCMV-VSV envelope construct, and the pLVX-Puro-ZsGreen1-c-Myc vector using Lipofectamine 2000 (Invitrogen, Cat. 11668027) and Opti-MEM medium (Gibco, Cat. 31985062). Cells were allowed to express the constructs for 48 h, with one media change at 24 h, before collecting the virus. Two wells were pooled, and the virus was concentrated using the LentiX Concentrator, following the protocol from Clontech (Cat. 631231) with >24 h incubation times at 4 °C. HeLa cells were infected in 6-well plates at between 55 and 75% confluency seeded at least 24 h in advance. Media were supplemented with 2 µg/mL final concentration Polybrene (Cat. TR-1003-G, Millipore, Burlington, MA, USA) to increase transduction efficiency. Media were changed after 24 h. After recovery, DMEM was supplemented with 2 μg/mL Puromycin Dihydrochloride (Gibco, Cat. A11138) for positive selection of transduced cells. Clonal populations were selected using limiting dilutions in 96-well plates and using light microscopy to determine wells with single cell clones and fluorescence microscopy to determine expression and localization of the fluorescently tagged protein. Well C5 of one of four 96-well plates contained a single colony of nuclear-expressed ZsGreen1, which was used for subsequent experiments. To enrich fluorescent cells after time in culture, stably transduced HeLa cells expressing ZsGreen1-c-Myc were isolated using a BD FACSAriaII Cell Sorter at Texas Children’s Hospital, part of the Baylor College of Medicine Cytometry and Cell Sorting Core (CCSC, Houston, TX, USA).

A retrovirus encoding OTUB1 in the Gateway-cloned pRetroX-IRES-DsRedExpress vector was generated in the same fashion as above with the appropriate retroviral packaging, capsid, reverse transcriptase, and integrase factors. Multiple myeloma cell line MM.1S (ATCC #CRL 1974) was transduced with empty pRetroX-IRES-DsRedExpress vector or the vector encoding OTUB1. Cells were sorted to 100% purity based on DsRedExpress expression with a BD FACSAria II sorter by the BCM Cytometry and Cell Sorting Core. Growth kinetics were analyzed by measuring live cells with acridine orange and propidium iodide staining (#CS2-0106, Nexcelom Bioscience, Lawrence, MA, USA) with a Cellometer Auto 2000 (Nexcelom Bioscience) in quadruplicates as previously published [36].

### 2.5. Proteasome Inhibitions

Cells were treated with 12.5 μM Lactacystin (Cayman Chemical, Cat. 70980) for 6 h to inhibit protein degradation by the proteasome prior to FACS analysis. The fast-acting drug Bortezomib (5 μM) was used for chase experiments (Cat. PS-341, Selleck Chemicals, Houston, TX, USA).

### 2.6. Chase Experiments

293T or HeLa cells were treated with 10 μg/mL Cycloheximide (Cat. 239764, Sigma-Aldrich, St. Louis, MO, USA) or 0.1% vol. DMSO and imaged or collected at specific time points. 

### 2.7. Transient Transfections

For flow cytometry: A stably transduced HeLa clone expressing ZsGreen1-c-Myc was transfected for flow cytometry with pLVX-USP13 and pcDNA-FBXW7 and their corresponding empty vectors using Lipofectamine 2000 Transfection Reagent (Invitrogen, Cat. 11668027) and OptiMEM I Reduced Serum Medium (Gibco, Cat. 31985070) in 24-well plates. They were incubated for 24 h with the transfection reagents, followed by medium change to fresh DMEM with supplements. The cells were then incubated for an additional 24 h prior to preparation for flow cytometry.

The deubiquitinase library was transfected into HeLa cell clones stably transduced with ZsGreen1-c-Myc in 24-well glass bottom plates with high-performance #1.5 cover glass (Cat. P24-1.5H-N, Cellvis, Mountain View, CA, USA). One construct was transfected per well. The media on the cells were changed to fresh, phenol red-free DMEM (Corning, Cat. 17-205-CV) with the same additions as the regular medium prior to transfection. A master mix of Lipofectamine 2000 and OptiMEM was created for 25 wells and distributed to the DNA/OptiMEM mixtures to avoid pipetting small volumes of Lipofectamine. Each well received 500 ng DNA, 1.75 μL Lipofectamine 2000, and 50 μL OptiMEM. Plates were imaged on the CellDiscoverer7 (Carl Zeiss Microscopy, White Plains, NY, USA), with incubation settings set for 37 °C, 5% CO_2_, and humidified. 

Analysis of deubiquitinases in control HeLa cells and 293T cells: Maternal HeLa cells were seeded in 6-well plates with coverslips (Electron Microscopy Sciences, Cat. 72204-01, #1.5) for imaging-based analysis (HeLa cells) or without coverslips for biochemistry (293T cells). Cells were transfected with individual deubiquitinase constructs at 2 μg/plate with Lipofectamine 2000 and OptiMEM. After 24 h, medium was changed, and cells were processed for analysis 24 h later. 

FLAG-OTUB1 for microscopy: FLAG-OTUB1 WT-pcDNA3.1 (Addgene, plasmid 118209) was transfected following the same protocol as the deubiquitinase hits [37]. 

OTUB1 depletion: Knockdown of OTUB1 was performed with pGIPZ constructs obtained from the CBASS core at Baylor College of Medicine. Cells were transfected with the empty pGIPZ vector as negative control or the following four OTUB1-specific shRNA constructs. After 24 h, medium was changed, and cells were harvested for analysis 72 h after the beginning of the transfection.

shRNA constructs:

− V2LHS_155121 (#21): CTGAAGATGACAACATCTA
− V3LHS_344822 (#22): GGACACTACGATATCCTCT
− V3LHS_638581 (#81): AGCGACTCCGAAGGTGTTA
− V3LHS_638586 (#86): AAGGAGTTGCAGCGGTTCA

### 2.8. Flow Cytometry

Cell analysis was performed using a Guava easyCyte flow cytometer (Luminex, Austin, TX, USA) at Texas Children’s Hospital (Houston, TX, USA). Stably transduced HeLa cells expressing ZsGreen1-c-Myc were transfected with pLVX-USP13, pcDNA-Fbxw7, pLVX, or pcDNA empty vector constructs 48 h before analysis or treated with 12.5 μM Lactacystin or DMSO control 6 h before analysis. Cells were trypsinized, counted, and resuspended in the proper amount of PBS + 2%FBS (250,000 cells/mL). Cells were filtered immediately before flow using 35 μm nylon mesh lids (Corning, Cat. 352235). Data were captured for approximately 13,000 cells, and FACS files were analyzed using FlowJo ver. 10.8.1 (Becton, Dickinson and Company, Ashland, OR, USA). 

### 2.9. Microscopy

**Figure 1:**
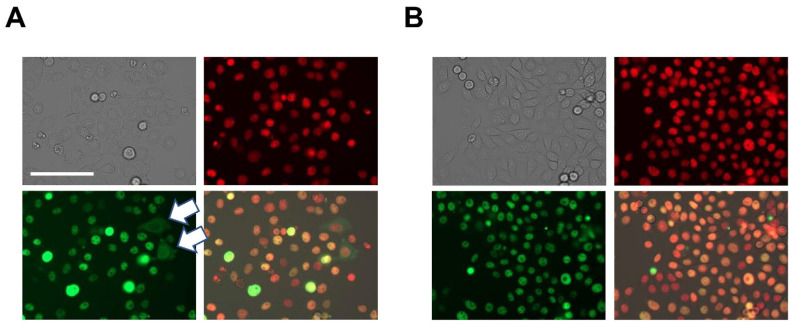
Selection of a fluorescent c-MYC reporter clone. HeLa cells were stably transduced with a lentiviral vector encoding the ZsGreen1-c-MYC fusion protein. The top left panels show brightfield, top right show nuclear counterstain, bottom left visualize ZsGreen1-c-MYC, and bottom right show the merged images. (**A**) The polyclonal population showed differences in expression levels of the fluorescent c-MYC protein. Several cells displayed extranuclear or even exclusive cytosolic expression of the fluorophore (white arrows). (**B**) Expansion of the cell clone used in this study with consistent nuclear expression of the fluorescent c-MYC fusion protein. Magnification was equal in both panels (20×), size bar represents 100 μm. Shown in red is the nuclear counterstain. Additional information is available in Figure S1.

Clonal analysis: ZsGreen1-c-MYC HeLa cells in Figure 1 were imaged using an EVOS FL microscope (Invitrogen, Software Revision 26133) with GFP settings. 

Deubiquitinase screen: HeLa clones stably transduced for ZsGreen1-c-Myc were plated in a 24-well glass bottom plate with high-performance #1.5 cover glass (Cellvis, Cat. P24-1.5H-N) and changed to phenol red-free media to avoid autofluorescence. The CellDiscoverer7 (Carl Zeiss) microscope was set up prior to transfection to reach 37 °C and 5% CO_2_ with humidity and allow the objectives to acclimate to that temperature. An imaging time course was set up using the ZEN Pro 3.1 software (Carl Zeiss) to take images at 4 evenly dispersed, non-overlapping locations per well. Images were taken in phase gradient contrast (PGC), the ZsGreen1 (ZsGr1) channel, and the TdTomato (TdTom) channel, which shares close excitation/emission spectrums with dsRedExpress. The Plan-Apochromat 20×/0.95 objective and 0.5× Tubelens were used for a total magnification of 10×. Images were taken every 2 h for 72 h with automatic focus. Background subtraction was performed using the same settings across all images, with a radius of 100 and smoothing beforehand. Images were analyzed using the ZEN Pro 3.1 software. ZsGreen1 segmentation was performed to collect data for individual cells. For each ZsGreen1 segmented cell, multiple measurements were analyzed, including area, ZsGreen1 and TdTomato mean intensity, and standard deviation of the channel. Data for the cells in each of the 4 images per well were compiled in Microsoft Excel, and the mean intensity of ZsGreen1 was plotted for each time point in a box-and-whisker plot (not shown). The plots and images were compared with the pRetroX-dsRedExpress Empty Vector plot to quantify a change in the mean ZsGreen1 intensity with expression of the deubiquitinase. The following criteria were used to determine “hits”: First, ZsGreen1 fluorescence at the 72 h time point in deubiquitinase-transfected cells had to be significantly higher than in cells transfected with an empty vector. As the cut-off for significance, we used a *p*-value < 1.0 × 10^−60^ by Student’s *t*-test (or <2.3 × 10^−33^ by the Wilcoxon rank-sum test). This *p*-value was chosen because it represents the knee point of all *p*-values when plotted on a logarithmic waterfall curve. Second, to account for outliers, we also calculated the median fluorescence in treated or mock-treated cells at the 72 h time point. Fluorescence in deubiquitinase-overexpressing cells had to be >1.2 fold higher by median than in control cells for inclusion. Third, to consider change over time, we measured the increment in the fluorescence change in the last 24 h of measurement. The increase in fluorescence during this period had to be greater in deubiquitinase-treated cells versus control cells. Plots and images were visually examined by two independent researchers to verify potential “hits” and exclude cell toxicity. Violin plots of the hits were created using the final time point in GraphPad Prism 9 (Version 9.2.0, GraphPad, San Diego, CA, USA). 

Cycloheximide time course: HeLa cell clones stably transduced with ZsGreen1-c-Myc cells were seeded in two quadrants of a Greiner CELLview 35 mm 4-compartment glass bottom dish (Cat. 627870), and tissue culture medium was added, including 24 h treatment with 10 μg/mL Cycloheximide (Sigma-Aldrich, Cat. 239764) or 0.1% vol. DMSO in phenol red-free DMEM (Corning, Cat. 17-205-CV). Cells were placed in the warmed and humidified CD7 microscope for imaging immediately. One field of view was used per compartment. Images were captured in ZsGreen1, TdTomato, and oblique every 5 min. TdTomato was captured for a parallel experiment. Plan-Apochromat 20×/0.7 objective with 0.5× Tubelens was used for a total magnification of 10×. Exposure times and display settings were kept constant between all images. The images were background subtracted (100 radius setting) following smoothing. Quantification was performed with the ZEN Pro 3.1 Suite (Zeiss).

Analysis of OTUB1 and endogenous c-MYC expression: Immunofluorescence for endogenous c-MYC, OTUB1, or FLAG-tagged OTUB1 was performed as follows: Cells were washed in PBS and fixed with 4% para-Formaldehyde (Thermo Scientific, Cat. 28906) for 15 min at room temperature. Then, 0.3% Triton X-100 (Cat. IB07100, IBI Scientific, Dubuque, IA, USA) permeabilization agent was added to all subsequent steps. Cells were then washed again and blocked in 5% BSA/PBST. Staining was performed at room temperature for one hour with 1:1000 anti-c-MYC (Cat. 13987, Cell Signaling Technology, Danvers, MA, USA), anti-OTUB1 (Cat. 270959, Abcam, Cambridge, MA, USA), or M2 anti-FLAG antibodies (Sigma-Aldrich, Cat. F1804) in 1% BSA/PBST. Actin was stained using fluorescent Phalloidin according to the manufacturer’s recommendations (Cell Signaling Technology, Cat. 12877). Following washing with PBST, appropriate Alexa Fluor 488 or Alexa Fluor 594 secondary antibodies were applied at 1:1000 for 45 min (Invitrogen), prior to washing in PBST with a final wash in PBS, and mounting using DAPI-containing medium (Invitrogen, Cat. P36935). For live cell microscopy, NucSpot Live 650 (Cat. 40082, Biotium, Fremont, CA, USA) was used as a nuclear counterstain in the presence of 25 μM Verapamil. Immunofluorescence was performed on a CD7 Zeiss microscope and quantified with the ZEN Pro 3.1 software. Quantifications of endogenous c-MYC were conducted at 20× magnification, counting more than 500 cells per condition in multiple visual fields. Violin plots of the anti-Myc fluorescent intensity were made in GraphPad Prism 9. The colocalization analysis was performed with the ZEN Pro 3.1 Suite following image capture in stacks with water immersion, deconvolution (nearest neighbor method, clip normalized), and background correction. Orthogonal projections are shown except for the colocalization mask, which represents a single z-stack of 290 nm thickness. The colocalization coefficient of c-MYC and OTUB1 was calculated based on [38] and consistently showed that >50% of c-MYC colocalized with the nuclear OTUB1 signal in multiple stacks and cells.

Proximity ligation assay: Interaction between c-MYC and OTUB1 was visualized using proximity ligation, which detects distances between two proteins of less than 40 nm. HeLa cells were transfected either with empty pcDNA3 vector or FLAG-OTUB1 vector. The assay was performed with anti-c-MYC (Cell Signaling Technology, Cat. 13987) at 1:500 concentration and M2 anti-FLAG antibodies (Sigma-Aldrich, Cat. F1804) at 1:50 using the Duolink kit (Sigma-Aldrich, Cat. DUO92101). The analysis was conducted with the ZEN Pro 3.1 Suite following image capture in 290 nm stacks over a vertical distance of 7.25 μm with water immersion, deconvolution (nearest neighbor method, clip normalized), and background correction. Orthogonal projections are shown to visualize the ligation signal that is otherwise dispersed on the z axis. 

### 2.10. Protein Extraction

Cells were carefully quantified prior to plating using a Cellometer Auto 2000 (Nexcelom Bioscience) with AOPI Staining (Nexcelom, Cat. CS2-0106). Cells were grown in duplicate in 2 wells of a 6-well plate and collected by washing 2× with ice-cold PBS. Cells were lysed using RIPA Lysis and Extraction Buffer (Sigma Aldrich, Cat. R0278) supplemented with Xpert protease inhibitor cocktail solution (GenDepot, Cat. P3100), manually scraped from the bottom of the wells, and transferred to Eppendorf tubes. The tubes were incubated on ice for 1 h with vortexing every 15 min and then spun down at 14,000 rpm for 30 min at 4 °C in a tabletop centrifuge. The protein layer was removed by pipetting, with care to avoid the lipid layer on top. Protein concentration was determined by the Bradford assay using BioRad Protein Assay Dye Reagent Concentrate (BioRad, Cat. 5000006) in a clear 96-well plate using BSA standards and read on a BioTek PowerWave XS plate reader with Gen5 software (version 2.09).

### 2.11. Western Blotting

Total protein was extracted and quantified as described above. Equal protein masses were diluted in 2× Laemmli Sample Buffer (Cat. 1610737, BioRad, Hercules, CA, USA) with reducing agent Beta-mercaptoethanol (Gibco, Cat. 21985-023) and denatured at 98 °C for 5 min prior to SDS-PAGE. Proteins were separated by SDS-PAGE under reducing conditions using AnykD Mini-PROTEAN TGX Stain-Free Precast Protein Gels (BioRad, Cat. 4568123) with Xpert 2 Prestained Protein Marker (Cat. P8503, GenDEPOT, Katy, TX, USA). Gels were run in Mini-PROTEAN TGX Stain-Free Precast Gels Electrophoresis System (BioRad, Cat. 1658004) with 1X Tris/Glycine/SDS (BioRad, Cat. 1610732) and then semi-dry transferred onto polyvinylidene difluoride (PVDF) membranes (BioRad, Cat. 1704156) for 7 min using a Trans-Blot Turbo Transfer Starter System (BioRad, Cat. 17001917). Then, PVDF membranes were blocked with 5% BSA in TBST. Western blot analysis was performed against c-Myc using c-Myc/N-Myc (D3N8F) Rabbit mAb (Cell Signaling Technology, Cat. 13987), against OTUB1 (Abcam, Cat. 270959), and with GAPDH (Abcam, Cat. 204481) as a loading control. Following incubation with HRP-conjugated goat anti-rabbit secondary antibody (Abcam, Cambridge, MA, USA, #ab6721 at 1:1000 dilution for 60 min), HRP-conjugated proteins were detected with the Clarity chemiluminescent substrate (BioRad, Cat. 1705060) and visualized using the BioRad ChemiDoc imaging system. Densitometry measurements were performed with the FIJI Image J suite [39].

### 2.12. RNA Quantification

Cells were grown in 2 wells of a 6-well plate and washed with ice-cold PBS prior to freezing dry pellets at −80 °C. RNA was collected using the RNeasy Plus kit (Qiagen, Cat. 74034) following the vendor’s protocol. The instruments and working area were cleaned to minimize RNase contamination, and DNA/RNA-free filtered pipet tips were used. Levels of endogenous *c-MYC* were measured compared to *GAPDH* or *ACTB* as a reference. RTqPCR was performed with a one-step kit (Thermo Scientific, Cat. 11736059) according to the manufacturer’s protocol on a BioRad CFX96 instrument. Relative gene expression was calculated based on the delta delta Ct method [40].

Primer sequences:

− F_GAPDH: GGAGCGAGATCCCTCCAAAAT
− R_GAPDH: GGCTGTTGTCATACTTCTCATGG
− F_MYC: GTCAAGAGGCGAACACACAAC
− R_MYC: TTGGACGGACAGGATGTATGC
− F_TP53: CAGCACATGACGGAGGTTGT
− R_TP53: TCATCCAAATACTCCACACGC
− F_BAX: CCCGAGAGGTCTTTTTCCGAG
− R_BAX: CCAGCCCATGATGGTTCTGAT
− F_CCND2: ACCTTCCGCAGTGCTCCTA
− R_CCND2: CCCAGCCAAGAAACGGTCC
− F_CCNE1: AAGGAGCGGGACACCATGA
− R_CCNE1: ACGGTCACGTTTGCCTTCC
− F_NPM1: GGAGGTGGTAGCAAGGTTCC
− R_NPM1: TTCACTGGCGCTTTTTCTTCA
− F_LDHA: TTGACCTACGTGGCTTGGAAG
− R_LDHA: GGTAACGGAATCGGGCTGAAT
− F_ACTB: CATGTACGTTGCTATCCAGGC
− R_ACTB: CTCCTTAATGTCACGCACGAT

### 2.13. Primary Multiple Myeloma Analysis

RNA-seq data from the CoMMpass study release IA10 were analyzed for expression levels in Fragments Per Kilobase of transcript per Million mapped reads (FPKM). The genome-wide expression of primary multiple myeloma cells in 811 patients was ranked based on the expression of 171 known and putative deubiquitinases. The survival analysis is based on the APEX study that examined treatment with bortezomib in relapsed/refractory multiple myeloma [41]. Statistics were calculated using the Mantel–Cox test using GraphPad Prism 9.

### 2.14. Statistical Validation

Each experiment showing the biochemical and imaging-based effects of OTUB1 on c-MYC expression was validated by more than three independent biological replicates. 

## 3. Results

### 3.1. Screen for Deubiquitinases Acting on c-MYC

In order to study the regulation of abundance of the c-MYC protein in human cells, we required a model system. Several c-MYC reporter constructs have been described in the literature [42]. Specifically, the fluorophore GFP has been fused to the N-terminus of c-MYC in a mouse model and revealed no apparent developmental or growth defects [43]. Applying this strategy to track levels of the c-MYC protein, we fused the fluorophore ZsGreen1 to the N-terminus of human c-MYC to track the abundance of the proto-oncogene. Stably transduced HeLa cells showed a remarkable variety in ZsGreen1-c-MYC expression, including multiple clones with extranuclear c-MYC. Further, the transduced cells had heterogeneous growth rates. We isolated single-cell clones and screened for consistent and exclusive nuclear expression of ZsGreen1-c-MYC. In addition, the growth kinetics of the selected cell clone used in subsequent screening experiments was comparable to unmodified control HeLa cells (Figure 1 and Figure S1). 

**Figure 2:**
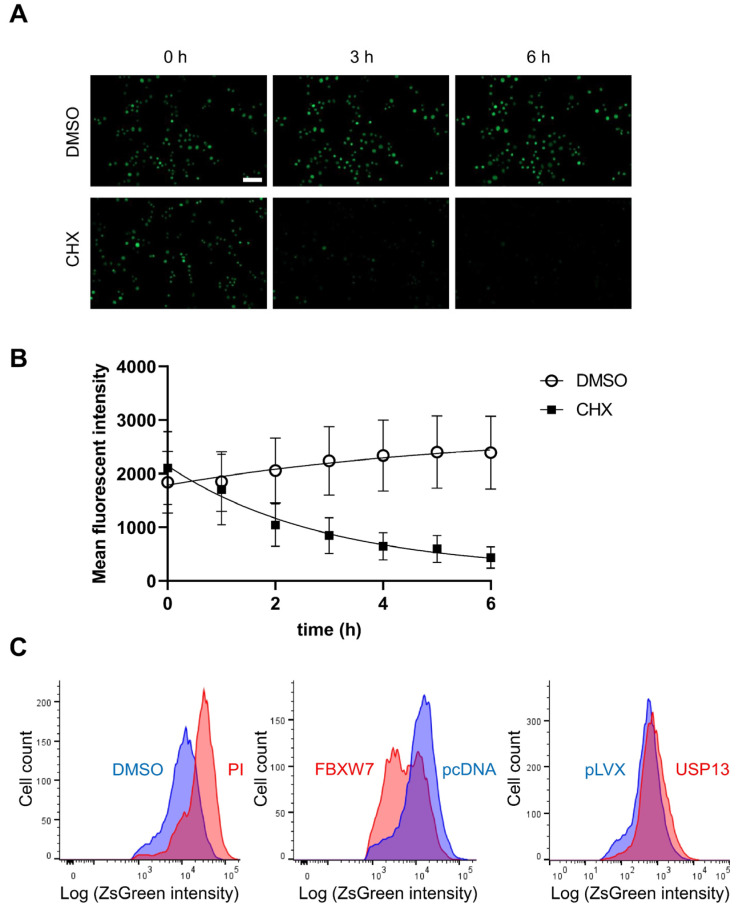
Proteolytic characterization of the ZsGreen1-c-MYC fusion protein. (**A**) Clonal HeLa cells that stably expressed ZsGreen1-c-MYC were analyzed for green fluorescence in the presence of solvent control (DMSO, 0.1% vol.) or equal volumes of the translation inhibitor Cycloheximide (CHX, 10 μg/mL). Scale bar represents 100 μm. (**B**) c-MYC is a short-lived protein. Similarly, the fluorescent fusion protein was also rapidly degraded with a half-life of 2.04 h based on quantification of epifluorescence microscopy. Data points denote mean values, error bars represent the standard deviations. The graph was modeled based on one-phase exponential decay (GraphPad Prism 9.2). The decay rate constant was calculated as 0.3402 (1/h). (**C**) FACS quantification of stable ZsGreen1-c-MYC reporter cells following 6 h treatment with proteasome inhibitor (PI) lactacystin (12.5 μM) compared to equal volume of solvent control DMSO, or following 48 h transfection with the ubiquitin ligase component FBXW7 (compared to empty vector pcDNA3), which accelerated removal of the fusion protein, or deubiquitinating enzyme USP13 (compared to empty vector pLVX), which reduced turnover of the c-MYC construct. The flow cytometry histogram x axis represents the logarithmic ZsGreen1 intensity, the y axis indicates cell counts. Shown is a representative of three biological replicates, with statistically significant differences (*p* < 10^−6^, Wilcoxon rank-sum test with Bonferroni correction after analysis of 10,000 cells).

Several elements of the ubiquitin–proteasome pathway control c-MYC. Cullin ligases promote the degradation of c-MYC using the FBXW7 substrate binding protein, which connects c-MYC to ubiquitin-conjugating enzymes and triggers its elimination [44,45,46,47]. On the opposite spectrum, several deubiquitinating enzymes, such as USP13, can rescue c-MYC from degradation [33]. To test whether the reporter cell line faithfully reflects the behavior of physiological c-MYC with respect to its ubiquitin–proteasome regulation, we first examined the half-life of the c-MYC protein in the stable HeLa cell clones (Figure 2A,B). Cycloheximide is a translation inhibitor that rapidly causes the depletion of the c-MYC protein due to its short half-life. We found that ZsGreen1-c-MYC had a half-life of approximately 2 h, indicating that the addition of a long-lived fluorophore at the N-terminus did not prohibit degradation of the c-MYC-containing fusion protein. This is in agreement with reports showing that GFP only slightly slows the turnover kinetics of c-MYC [42]. The half-life of endogenous c-MYC ranges between 30 and 120 min in most of the published literature and in our own studies. 

Next, we treated the reporter cell line with the proteasome inhibitor lactacystin. Proteasome inhibition significantly increased c-MYC levels in the nucleus (Figure 2C). To test the impact of individual ubiquitin ligases and deubiquitinating proteases, we transfected the reporter cell line with the Cullin ligase component FBXW7. As expected, overexpression of FBXW7 reduced ZsGreen1-c-MYC levels. In contrast, overexpression of the deubiquitinating enzyme USP13 increased the levels of ZsGreen1-c-MYC (Figure 2C). These flow cytometry studies indicate the fluorophore-linked c-MYC in the reporter cell line behaves similarly to the endogenous c-MYC protein with regard to degradation regulation.

**Figure 3:**
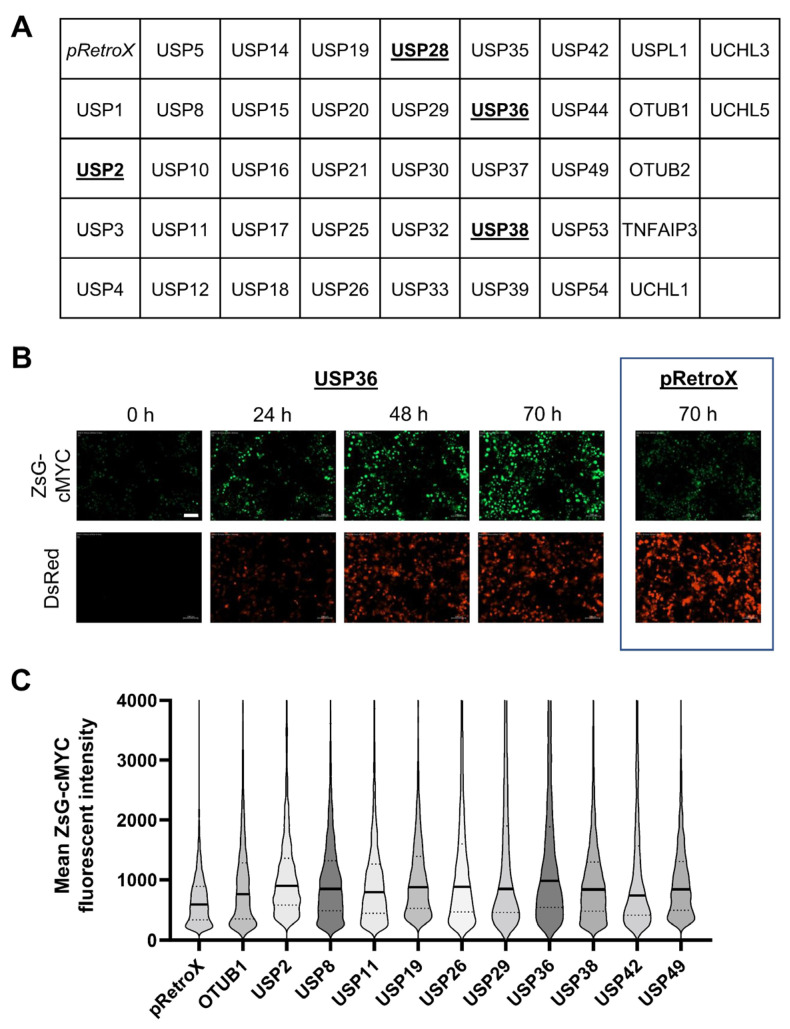
Screen for altered ZsGreen1-c-MYC protein levels following overexpression of deubiquitinases. (**A**) The stable HeLa reporter cell line expressing ZsGreen1-c-MYC was transfected with an empty vector (pRetroX-IRES-DsRedExpress) or with 1 of 41 deubiquitinases, cloned into pRetroX-IRES-DsRedExpress. Deubiquitinases with previously published activity on c-MYC are underscored. Cells were transfected with the deubiquitinase-encoding vectors at the beginning of the experiment, and images were taken every 2 h. Additional information on the screen is provided in Figures S2 and S3. (**B**) Microscopy time course of the known c-MYC-specific deubiquitinase USP36 with expression of the ZsGreen1-c-MYC fusion protein (top panel) and the USP36- and dsRedExpress-encoding vector (bottom panel). Cells transfected with the empty control vector pRetroX-IRES-DsRedExpress are shown on the right (70 h time point depicted). The scale bar represents 100 μm. (**C**) Quantification of ZsGreen1-c-MYC fluorescence at time point 72 h (median in bold). Multiple visual fields and a minimum of 2400 cells were analyzed per transfected cell batch. Eleven deubiquitinating enzymes increased ZsGreen1-c-MYC levels, including eight new and three previously published candidates (USP2, USP36, and USP38). Fluorophore bleed-through was excluded prior to analysis. The minimum difference in ZsGreen1-c-MYC expression corresponds to *p* < 2.3 × 10^−33^ (Wilcoxon rank-sum test with Bonferroni correction) at the 72 h time point and showed a trajectory of increase compared to the empty vector control over time.

To identify c-MYC-specific deubiquitinases, we opted for an overexpression screen, as loss of essential deubiquitinases might decrease c-MYC expression as a consequence of non-specific cellular toxicity. Further, expression of several deubiquitinase enzymes is cell type dependent, and knockdown or CRISPR studies will miss proteases that are not expressed in the investigated cell type. We hypothesized that increasing the expression of a c-MYC-specific deubiquitinase should increase c-MYC levels, which is unlikely to reflect non-specific cell toxicity. We transfected the reporter cell line individually with an empty control plasmid or 41 plasmids encoding different human deubiquitinases that are available through the Ultimate ORF and human ORFeome libraries (Figure 3A). These proteases represent four different families of deubiquitinases [6,31]. 

Using live cell microscopy over the course of 72 h, we tracked transfection efficiency with a DsRedExpress reporter in the deubiquitinase-encoding plasmid. We quantified ZsGreen1-c-MYC levels with automatic image acquisition every two hours (Figure 3B). Using this approach, we identified 11 potential stabilizers of c-MYC, including 8 new and 3 known c-MYC-specific deubiquitinating proteases, USP2, USP36, and USP38 [34,48,49] (Figure 3C). One previously reported c-MYC-specific protease, USP28 [18], failed to significantly increase ZsGreen1-c-MYC in the reporter cell line (Figures S2 and S3).

### 3.2. Otubain-1 Stabilizes c-MYC Protein Levels

**Figure 4:**
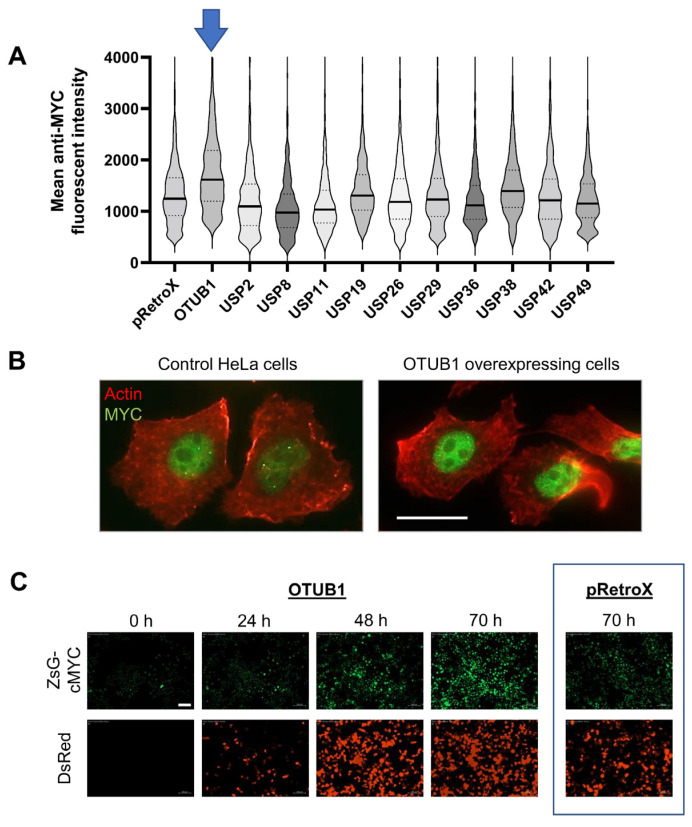
Validation of deubiquitinase effects on endogenous c-MYC. (**A**) Control HeLa cells were individually transfected for 48 h with the eleven deubiquitinating enzymes from the previous screen, and expression of endogenous c-MYC was quantified by immunofluorescence microscopy. Compared to the empty pRetroX-IRES-DsRedExpress control vector, only OTUB1 overexpression increased the levels of endogenous c-MYC substantially (*p* < 1.76 × 10^−28^ by Wilcoxon rank-sum test with Bonferroni correction; median shown in bold). More than 500 cells and multiple visual fields were analyzed per condition. (**B**) Example showing 50× magnification of control HeLa cells transfected with empty vector (left) and stained for Actin (red) or endogenous c-MYC (green). OTUB1 overexpression (right) increased c-MYC levels. Scale bar denotes 20 μm. (**C**) Microscopy time course from the original screen in the stable ZsGreen1-c-Myc HeLa cells with expression of ZsGreen1-c-Myc fusion protein in the top panel and the OTUB1- and dsRedExpress-encoding vector in the bottom panel. Stable ZsGreen1-c-Myc cells transfected with the pRetroX-IRES-DsRedExpress empty vector are shown on the right (70 h time point depicted).

As the ZsGreen1-c-MYC reporter construct involves a fusion protein, proteases might interact with the fluorophore domain instead of the c-MYC moiety. To exclude this possibility and isolate proteases with a specific effect on c-MYC at physiological expression levels, we next determined how the proteases impacted endogenous c-MYC in unmodified HeLa cells. We transfected native HeLa cells individually with the 11 identified deubiquitinase candidates and compared the protein levels of the proto-oncogene c-MYC by immunofluorescence microscopy. Cells transfected with an empty plasmid served as a reference. Using this approach, we found robust upregulation of c-MYC levels with only 1 of our 11 proteases: Otubain-1 [50] (Figure 4). OTUB1 is the founding member of the OTU (ovarian tumor domain) family of deubiquitinases and has been shown to regulate several cancer-related pathways, including TGFβ signaling, DNA repair, TP53, mTORC1, and RAS signaling [51]. Further, OTUB1 is one of the most abundant deubiquitinases in cells [52] and can act as an oncogene in several types of cancers, including lung, breast, gastrointestinal, and prostate cancers, as well as glioma [9]. However, OTUB1 has thus far not been identified as a regulator of c-MYC, a proto-oncogene that is dysregulated in up to 70% of all cancers [25]. 

**Figure 5:**
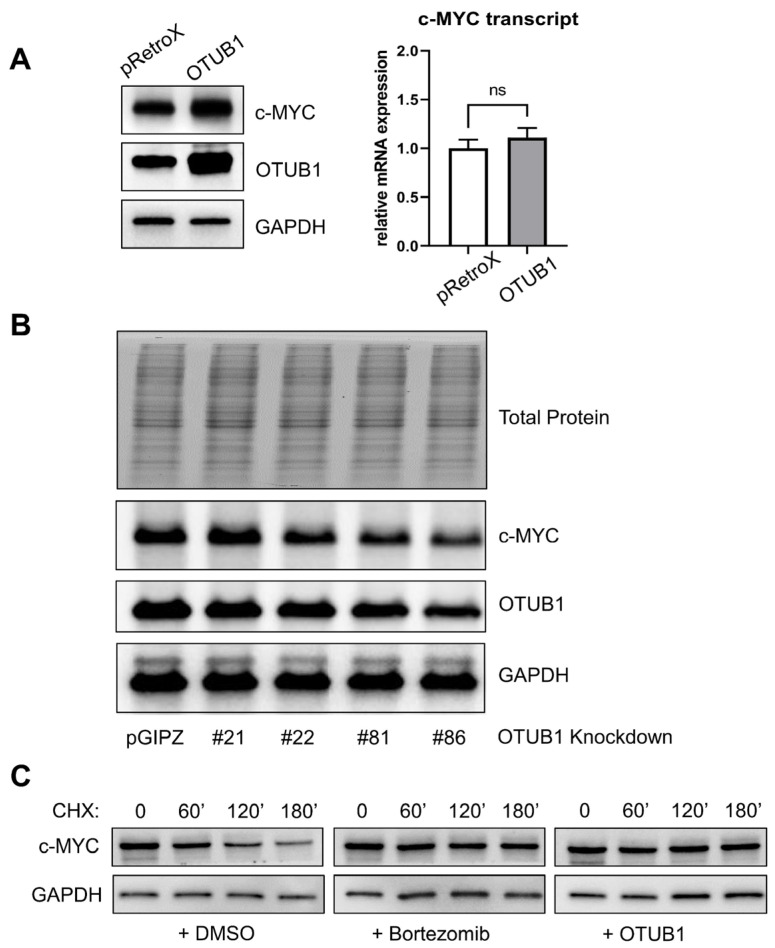
Biochemical validation of the effects of OTUB1 on c-MYC. (**A**) Forty-eight hours post-transfection with OTUB1, 293T cells showed slightly but consistently increased c-MYC levels (up to 1.5-fold increased by densitometry compared to GAPDH). This increase was not observed at the mRNA level, indicating that OTUB1 acts on c-MYC at the protein level. (**B**) Knockdown of OTUB1 by transfection with shRNA vectors for 72 h decreased c-MYC levels in 293T cells. We observed the strongest knockdown effect using construct #86 (V3LHS_638586) with a reduction in OTUB1 of 50% and a comparable 50% reduction in endogenous c-MYC by densitometry compared to GAPDH. The empty pGIPZ vector was used as a reference. Total protein visualized by trihalo staining. (**C**) Cycloheximide (CHX, 10 μg/mL) chase in minutes. Endogenous c-MYC was rapidly depleted following translation inhibition in 293T cells. This effect was counteracted by proteasome inhibitors (Bortezomib, 5 μM). Cells transfected with OTUB1 also show a marked stabilization of c-MYC in the chase. Transfections were performed 48 h prior to the experiment and included empty vector control pRetroX-IRES-DsRedExpress for the cells analyzed in the left and middle panels. Detailed densitometry readings of the uncropped immunoblots are provided as addendum in Figure S7.

In 293T cells, we used immunoblotting to confirm that c-MYC protein levels are increased by OTUB1 overexpression and that this effect is not driven by transcriptional upregulation of c-MYC (Figure 5A). Further, knockdown of OTUB1 reduced c-MYC levels by up to 50%, indicating that the dosage of OTUB1 regulates c-MYC levels (Figure 5B). 

**Figure 6:**
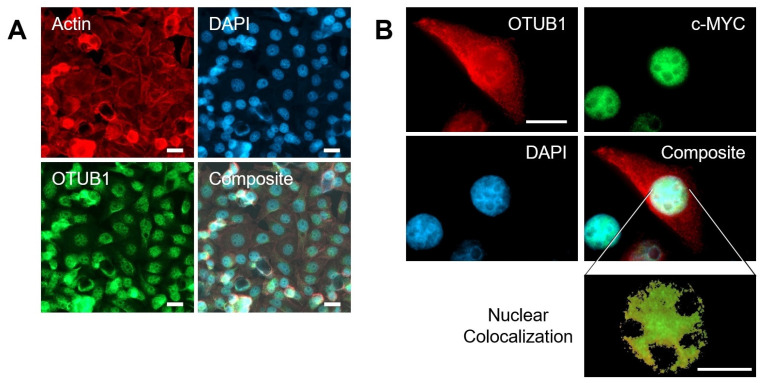
OTUB1 and c-MYC colocalize. (**A**) Immunofluorescence of endogenous OTUB1 confirmed nuclear expression of the deubiquitinase in HeLa cells. Cells were stained for Actin, OTUB1, and the nuclear marker DAPI. Magnification 50×, bar represents 20 μm. (**B**) Colocalization experiment with HeLa cells transfected with FLAG-tagged OTUB1. The tag allowed the simultaneous detection of OTUB1 with the anti-FLAG antibody in combination with an endogenous c-MYC antibody to visualize both proteins. Inset showing a 100× magnification of a single z-stack of the nucleus depicts c-MYC signal in green and OTUB1 in red. Both proteins robustly colocalized in this compartment. Bar represents 10 μm.

To test whether OTUB1 interferes with c-MYC degradation, as expected for a specific deubiquitinating protease, we next examined c-MYC protein turnover. In 293T cells, endogenous c-MYC has a half-life of approximately 120 min in Cycloheximide chase experiments. This decay is mostly driven by proteasomal degradation and halted when proteasome inhibitors are applied. Overexpression of OTUB1 similarly stabilized c-MYC in the presence of Cycloheximide. This result indicates that OTUB1 increases c-MYC protein levels by preventing its turnover (Figure 5C). 

c-MYC is immediately transported into the nucleus upon translation. Here, c-MYC binds to DNA, becomes ubiquitinated following activation, and is degraded by the proteasome [26,28]. For OTUB1 to act on c-MYC, the protease also needs to be present in the nucleus. To examine the localization of OTUB1 in native HeLa cells, we performed immunofluorescence microscopy for OTUB1 and found it to be cytoplasmic and nuclear, indicating a shared subcellular residence with c-MYC (Figure 6). This result is consistent with studies showing that OTUB1 can enter the nucleus following phosphorylation [53]. Further, combined staining for OTUB1 and c-MYC showed substantial colocalization of these two proteins in the nucleus, with >50% overlap in single z-stacks. To further validate this finding, we next performed highly sensitive proximity ligation assays, which also suggested that a physical association between OTUB1 and c-MYC exists (Figure S4). 

**Figure 7:**
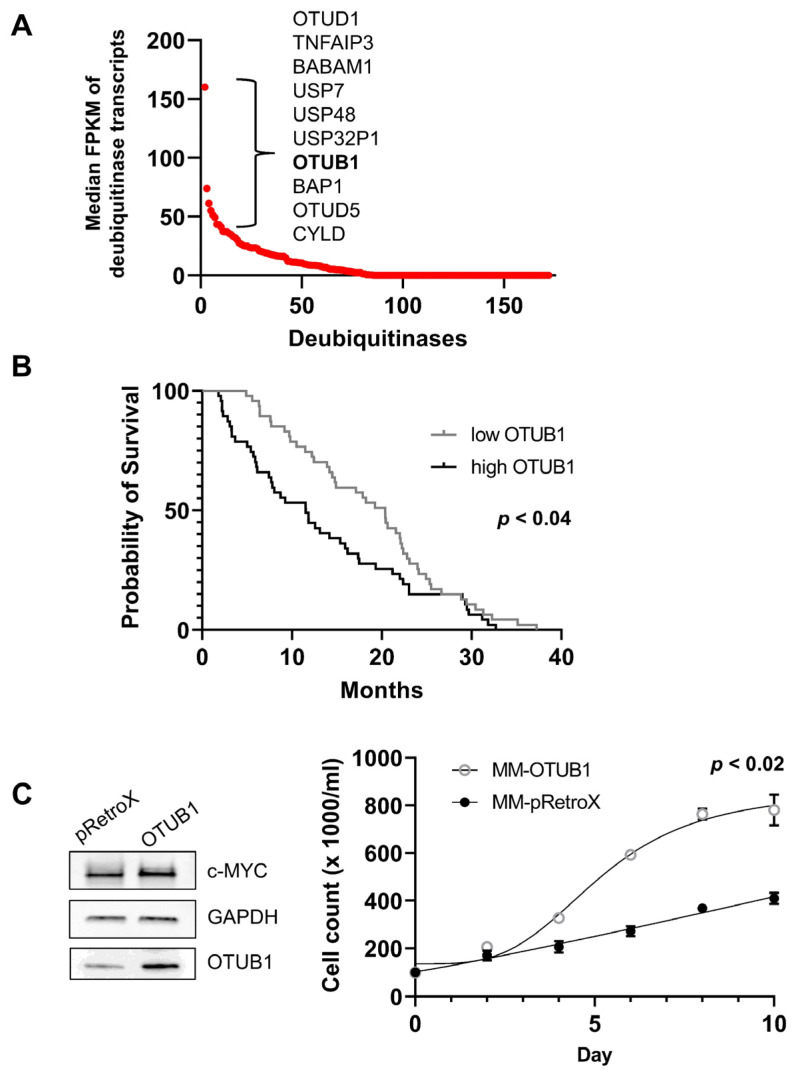
OTUB1 has oncogenic potential in multiple myeloma. (**A**) Analysis of expression levels of deubiquitinases in primary multiple myeloma cells of 811 patients. OTUB1 was among the top 10 enzymes of this class expressed in these cancer cells. (**B**) Multiple myeloma patients with elevated OTUB1 levels (top versus bottom quartile) showed worse clinical outcomes based on clinical data from the APEX study [41]. Mantel–Cox analysis of the Kaplan–Meier plot indicated that OTUB1 increases the oncogenic potential of the c-MYC-dependent cancer multiple myeloma (*p* < 0.04). (**C**) Left panel: Immunoblot of MM.1S cells transduced with empty pRetroX control vector (left lane) or the OTUB1-encoding vector (right lane). Endogenous c-MYC was 1.5-fold increased based on densitometry in OTUB1-overexpressing cells (using GAPDH as a reference, not shown). Right panel: OTUB1-transduced MM.1S cells significantly outgrew cells transduced with the empty vector (*p* < 0.02 by paired two-sided Student’s *t*-test after day 4). Uncropped immunoblots are provided as addendum in Figure S8.

OTUB1 has been reported to have oncogenic potential in several solid tumors, facilitating epithelial-mesenchymal transition in colorectal cancer cells and glioma cells, and inducing metastatic spread in esophageal squamous cell carcinoma [9,51]. To examine whether OTUB1 expression has a physiological impact on c-MYC-addicted cancers, we analyzed its expression in multiple myeloma. Multiple myeloma, a malignancy of terminally differentiated plasma cells, is the second most common hematopoietic cancer and requires high c-MYC activity for progression, growth, and dissemination [54]. We quantified and ranked the mRNA expression of all known deubiquitinating proteases in multiple myeloma cells of 811 patients, made available by the Multiple Myeloma Research Foundation [55]. OTUB1 was highly expressed in primary multiple myeloma cells (Figure 7A). The only other deubiquitinase with higher expression levels that was previously shown to increase c-MYC levels was USP7 (HAUSP). However, USP7 may elevate c-MYC predominantly by acting on the transcription of this proto-oncogene, rather than stabilizing the c-MYC protein [56]. To assess whether OTUB1 has an effect as a tumor suppressor or an oncogene in multiple myeloma, we examined the survival of patients based on the expression of OTUB1 in the cancer cells. Patients with a high expression of the deubiquitinase showed significantly worse clinical outcomes (Figure 7B and Figure S5).

To test whether upregulation of c-MYC following OTUB1 overexpression has a physiological impact on multiple myeloma, we transduced the MM.1S cell line either with an empty retroviral vector or with a vector encoding OTUB1. Cells overexpressing OTUB1 showed superior growth kinetics and higher c-MYC protein levels, suggesting that elevated OTUB1 has oncogenic potential in this model (Figure 7C). In addition, the transcription of known c-MYC target genes in MM.1S cells was elevated in OTUB1-overexpressing cells (Figure S6).

## 4. Discussion

In this study, we sought to investigate elements of the ubiquitin–proteasome system that regulate the stability of c-MYC. c-MYC is a short-lived transcription factor that is generally highly expressed, and its quantity is carefully maintained at a temporal and spatial level [26]. c-MYC serves as a proto-oncogene in many cancers [25]. While rarely mutated in a way that alters the protein sequence [57], c-MYC is often found dysregulated in malignancies through altered expression levels and functions [22,58,59,60]. 

Prior screens for c-MYC-specific deubiquitinases have generally involved knockout or knockdown conditions. In earlier studies, the absence of a specific deubiquitinase reduced c-MYC levels. However, c-MYC is also reduced in cells that are generally sick or show impaired proliferation. Since several deubiquitinating proteases are essential [61], the lack of c-MYC expression might reflect the toxicity of the gene depletion strategy. Furthermore, several deubiquitinases show tissue-specific expression and may be missed in depletion screens using a single cell line. Instead, we opted for a deubiquitinase overexpression screen, using elevated c-MYC levels as our primary readout.

Deubiquitinases can rescue a protein slated for degradation by removing the ubiquitin chain from the target, rendering it invisible to the proteasome. We describe a genetic screen that identifies OTUB1 as a novel deubiquitinase that can stabilize c-MYC. OTUB1 alters endogenous c-MYC levels, as well as a transgenic reporter construct, indicating that it operates at the protein level and not through specific transcriptional regulation (Figure 5A).

The screen revealed a higher sensitivity of various deubiquitinases using a transgenic fluorophore fusion protein of c-MYC compared to endogenous c-MYC. Several known and potential c-MYC-specific proteases stabilized the c-MYC reporter construct but showed no effect when examining endogenous c-MYC levels in HeLa cells. Given its importance, c-MYC turnover is controlled by multiple ubiquitin ligases and deubiquitinases [62], and we suspect a high degree of redundancy under physiological conditions. Overexpression of the c-MYC protein through the reporter construct may challenge the steady state sufficiently to show a phenotype for 11 of the deubiquitinases. MYC protein levels are approximately 11-fold higher in the ZsGreen1-c-MYC reporter cell line compared to unmodified HeLa cells. Under normal conditions, a substantial fraction of c-MYC is bound to other proteins to form transcriptional complexes and may not be readily accessible to deubiquitinases. We suspect that an excess of free c-MYC in the reporter cell line facilitates binding to these proteases, rendering this system more sensitive as a screening tool than wildtype HeLa cells. This is also confirmed by the stabilization of the c-MYC fluorophore by three of its four known deubiquitinases (USP2, USP36, USP38). The previously identified enzyme that failed to increase c-MYC, USP28, requires a protein co-factor for interaction with c-MYC [18]. An isolated increase in USP28, in the absence of an elevated co-factor, might therefore limit the effect of the deubiquitinase in this overexpression system.

Only OTUB1 stabilized c-MYC at endogenous levels in our experimental model system. OTUB1 is an essential protein in mammals, has oncogenic properties in several solid tumors, and regulates transcriptional and signaling pathways involved in cancer progression and proliferation [51,63]. Our results indicate that OTUB1 also regulates the central proto-oncogene c-MYC. OTUB1 activity involves two distinct functions. First, it can remove lysine 48-linked ubiquitin chains from a substrate [64]. These chains are critical for the recognition of target proteins by the proteasome. Second, OTUB1 can prevent ubiquitination altogether by inhibiting the ubiquitin transfer reaction [65,66]. Future research will be necessary to examine how OTUB1 stabilizes the c-MYC protein, how these two proteins interact, and in which cellular sub-compartment c-MYC is targeted by the deubiquitinase. 

The identification of OTUB1 is relevant, because unlike most other previously reported c-MYC-specific deubiquitinases, it is expressed at high levels [51]. OTUB1 is, therefore, a protease capable of surveying the substantial quantities of the c-MYC protein that are present in physiological and malignant tissues. In primary multiple myeloma cells, the OTUB1 transcript is expressed within the top 5% of all deubiquitinases (Figure 7A), and in HeLa cells, its expression level ranks in the top 12%. Furthermore, we and others show that OTUB1 is expressed in the nucleus and colocalizes with c-MYC [53]. 

Multiple myeloma is a plasma cell-derived cancer, which requires c-MYC activity to progress and disseminate [67]. Our analysis indicates that OTUB1 acts as an oncogene in this c-MYC-dependent cancer type and that overexpression of OTUB1 increases c-MYC protein levels. Given that deubiquitinases are druggable enzymes [68,69], OTUB1 may be a relevant target for the development of new inhibitors.

## 5. Conclusions

In this study, we used stably expressed, fluorophore-tagged c-MYC and overexpression of a deubiquitinase library in an imaging-based screen to uncover unknown c-MYC regulators in a model cell line. We identified the deubiquitinating enzyme OTUB1 as a novel stabilizer of c-MYC at the protein level. Both OTUB1 and c-MYC have been previously identified as potential oncogenes. However, OTUB1 has not been implicated in enhancing c-MYC activity before. This finding is significant because direct pharmacological targeting of c-MYC has proven challenging. Deubiquitinases, on the other hand, are perfectly targetable proteases, and several therapeutic inhibitors are currently in development. We illustrate a relationship between high OTUB1 expression and decreased survival in multiple myeloma, a c-MYC-dependent cancer, and confirm the ability of OTUB1 to stabilize c-MYC in a multiple myeloma cell line. The identification of OTUB1 as a stabilizer of c-MYC reveals a new potential target for treating c-MYC in cancers.

## Data Availability

Select plasmids used in this study have been deposited with Addgene under ID #180278 and #180279.

## Supplementary Materials

The following supporting information can be downloaded at: www.mdpi.com/article/10.3390/cancers14030806/s1, Figure S1: Characterization of ZsGreen1-c-MYC HeLa cell clone; Figure S2: Raw data from the imaging-based screen; Figure S3: Tabular results from the imaging-based screen; Figure S4: Interaction study for c-MYC and OTUB1; Figure S5: Survival plot based on OTUB1 expression (CoMMpass study); Figure S6: OTUB1 increases c-MYC activity; Figure S7: Uncropped immunoblot for Figure 5; Figure S8: Uncropped immunoblot for Figure 7.
