# Supplementary material for: Imaging-Based Screening of Deubiquitinating Proteases Identifies Otubain-1 as a Stabilizer of c-MYC
Source: Cancers (Basel). 2022 Feb 4;14(3):806. doi: 10.3390/cancers14030806 (PMC8833929; doi:10.3390/cancers14030806)
Supplement: Supplementary file 1 [file cancers-14-00806-s001.zip › cancers-1547043-supplementary.pdf]

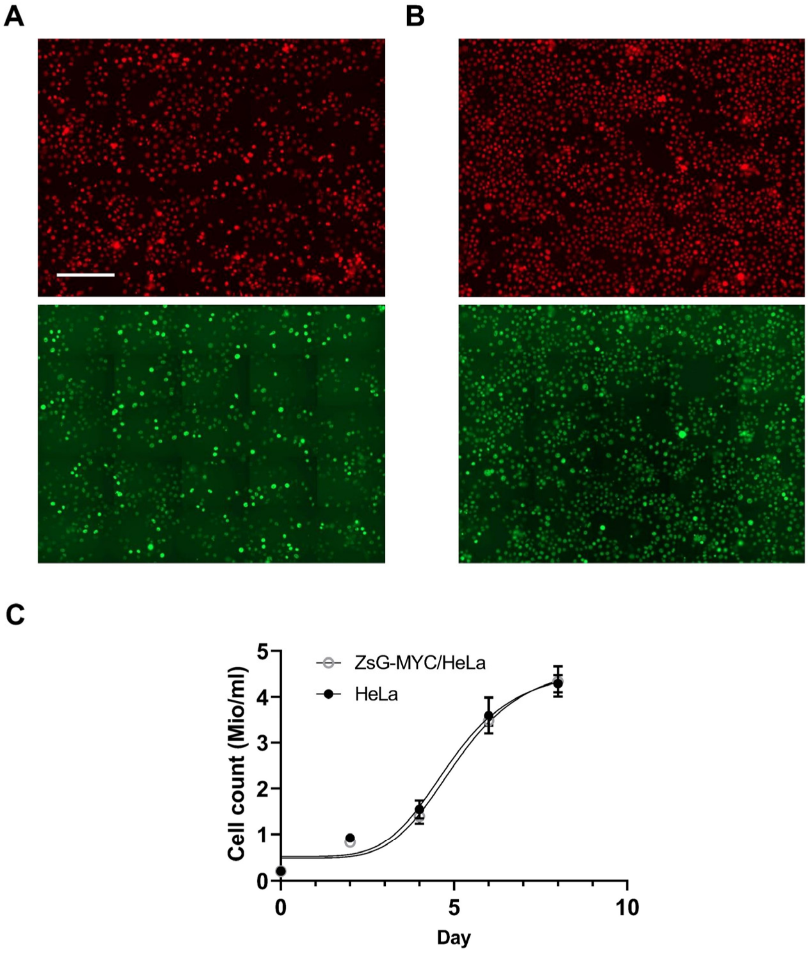

Figure S1. Selection of a fluorescent c-MYC reporter clone. (**A**) The polyclonal population showed higher variability in expression levels of the fluorescent c-MYC protein. (**B**) Expansion of the cell clone used in this study shows less variable c-MYC levels. Panomaric image of 25 stitched visual fields with equal magnification in both panels (20×). Size bar represents 200 µm. Shown in red is the nuclear counterstain. (**C**) Growth curves of equally seeded unmodified HeLa cells compared to the selected ZsGreen1-c-MYC clone used in this study showed no significant difference in proliferation. Live cells were counted with a Cellometer Auto 2000.

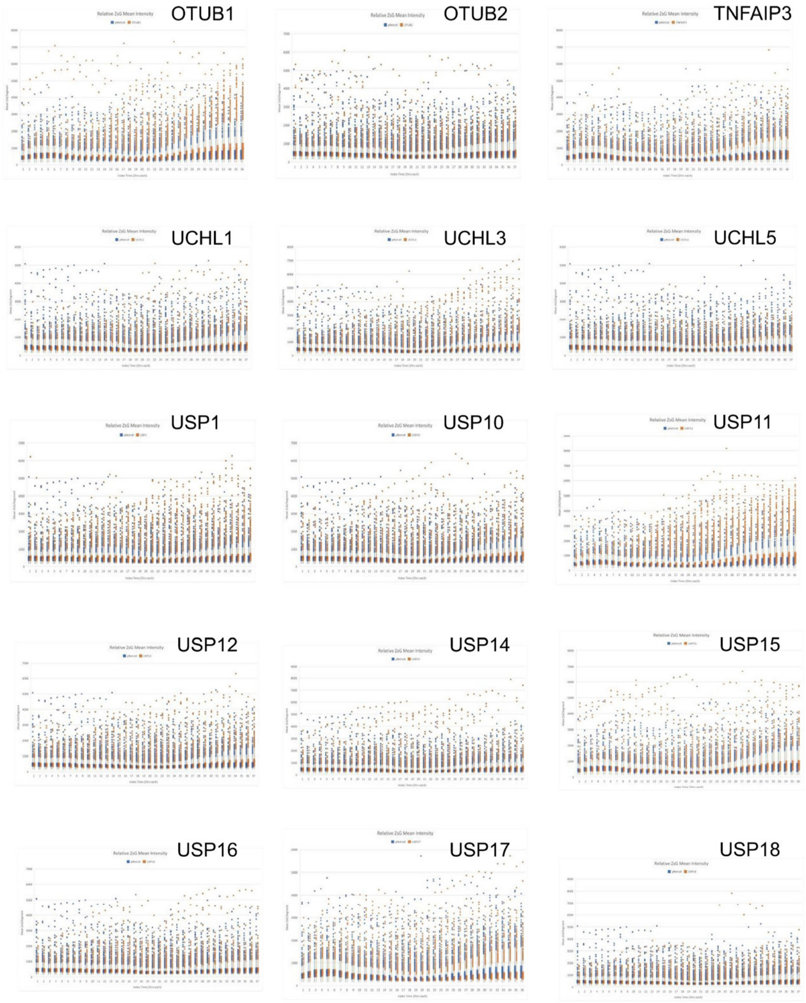

Figure S2. Raw data showing fluorescence over time in ZsGreen1-c-MYC transduced HeLa cells. Images were taken every two hours. Cells transfected with an empty control vector are indicated in blue, cells transfected with deubiquitinase are indicated in orange in this box and whisker plot. Continued on the next page.

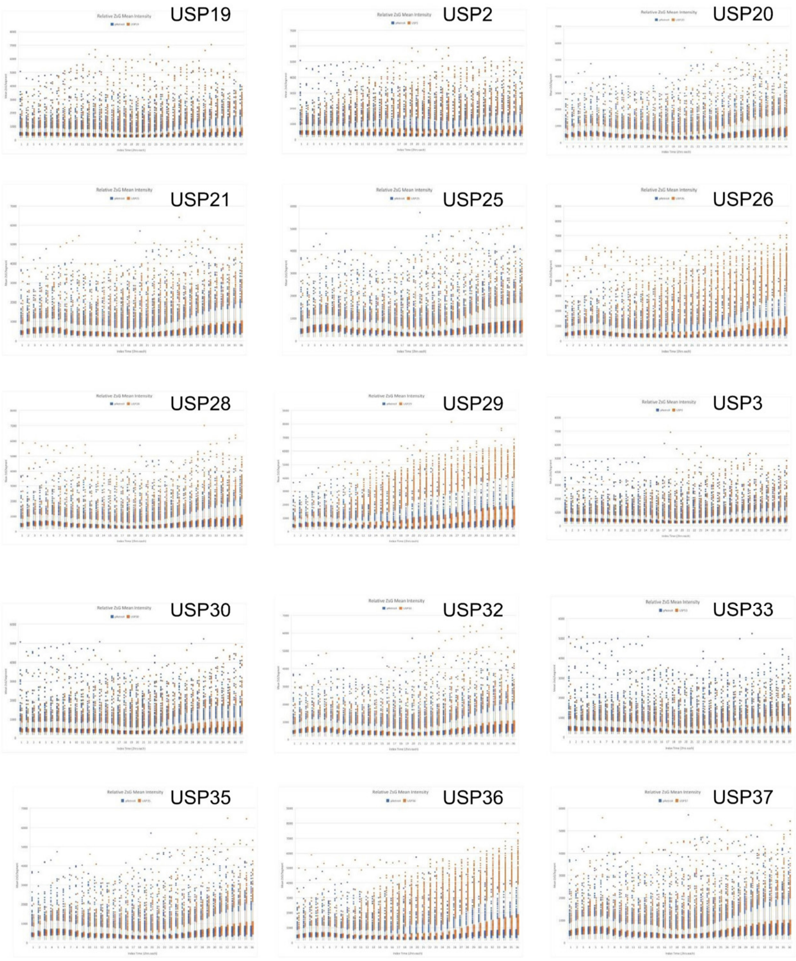

Figure S2. Raw data showing fluorescence over time in ZsGreen1-c-MYC transduced HeLa cells. Images were taken every two hours. Cells transfected with an empty control vector are indicated in blue, cells transfected with deubiquitinase are indicated in orange in this box and whisker plot. Continued on the next page.

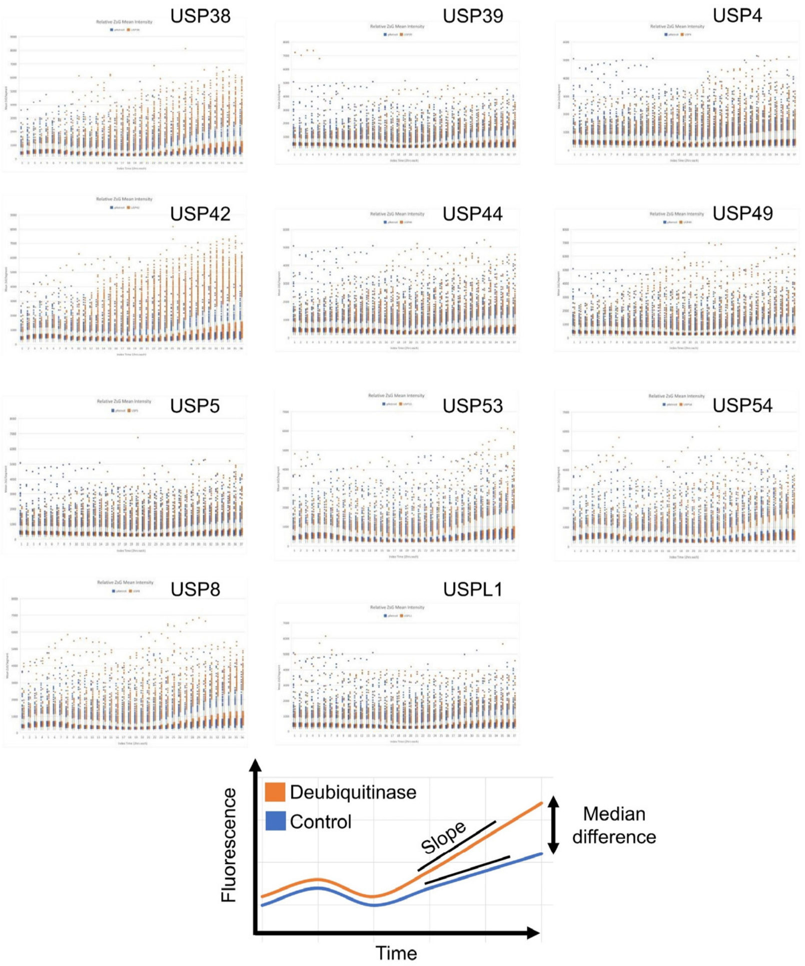

Figure S2. Continuation of raw data showing fluorescence over time in ZsGreen1-c-MYC transduced HeLa cells. Images were taken every two hours. Cells transfected with an empty control vector are indicated in blue, cells transfected with deubiquitinase are indicated in orange in this box and whisker plot. Bottom graph: Hits were considered based on the p-values of difference at the final time point ($p < 1.0 \times 10^{-60}$, Student's two-sided t-test), based on the median difference at the final time point (>1.2), and based on steeper increases in deubiquitinase-transfected cells over time.

DUBs	p-values	Median increase	Diff. increment
OTUB1	4.74E-74	1.284446	1.02749
OTUB2	1E-07	1.053986	1.096087
TNFAIP3	0.120365	0.945172	1.070578
UCHL1	2.09E-21	1.207522	1.205355
UCHL3	1.51E-35	1.242053	1.229127
UCHL5	5.76E-08	0.860149	1.004396
USP1	1.8E-40	1.19821	1.152237
USP10	2.22E-34	1.14864	1.079632
USP11	2.89E-94	1.340327	1.048355
USP12	3.74E-54	1.299338	1.275638
USP14	7.35E-26	1.26642	1.159934
USP15	4.78E-31	1.1723	1.043197
USP16	0.007044	1.053992	1.10992
USP17	1.72E-83	0.677276	0.834662
USP18	3.27E-07	1.098736	1.149476
USP19	1.75E-91	1.477953	1.13738
USP2	6.52E-98	1.513139	1.131893
USP20	9.96E-11	1.070137	1.134302
USP21	1.93E-29	1.158393	1.088589
USP25	7.99E-05	1.06361	1.067446
USP26	5.6E-156	1.485287	1.37577

DUBs	p-values	Median increase	Diff. increment
USP28	6.25E-41	1.203185	1.12468
USP29	3.4E-183	1.432596	1.053571
USP3	6.87E-34	1.284055	1.149392
USP30	2.53E-58	1.245313	1.082029
USP32	1.45E-35	1.181676	1.144712
USP33	1.33E-12	0.826399	0.937153
USP35	1.34E-48	1.237665	1.2869
USP36	1.9E-221	1.656369	1.51051
USP37	1.41E-19	1.129496	0.988773
USP38	3.9E-107	1.412202	1.089693
USP39	3.42E-29	1.16414	0.949264
USP4	0.000139	1.034202	1.034358
USP42	1.8E-128	1.243923	1.214917
USP44	9.22E-23	1.204438	1.02948
USP49	3.97E-65	1.422138	1.233179
USP5	3.01E-27	1.182744	1.114272
USP53	3.55E-20	1.121586	1.330516
USP54	0.016187	0.932652	1.129353
USP8	6.3E-117	1.430934	1.173818
USPL1	9.87E-51	1.393835	1.197638

Figure S3. Tables summarizing the results of the screen of ZsGreen1-c-MYC transduced HeLa cells. Deubiquitinases considered positive hits are indicated with arrows.

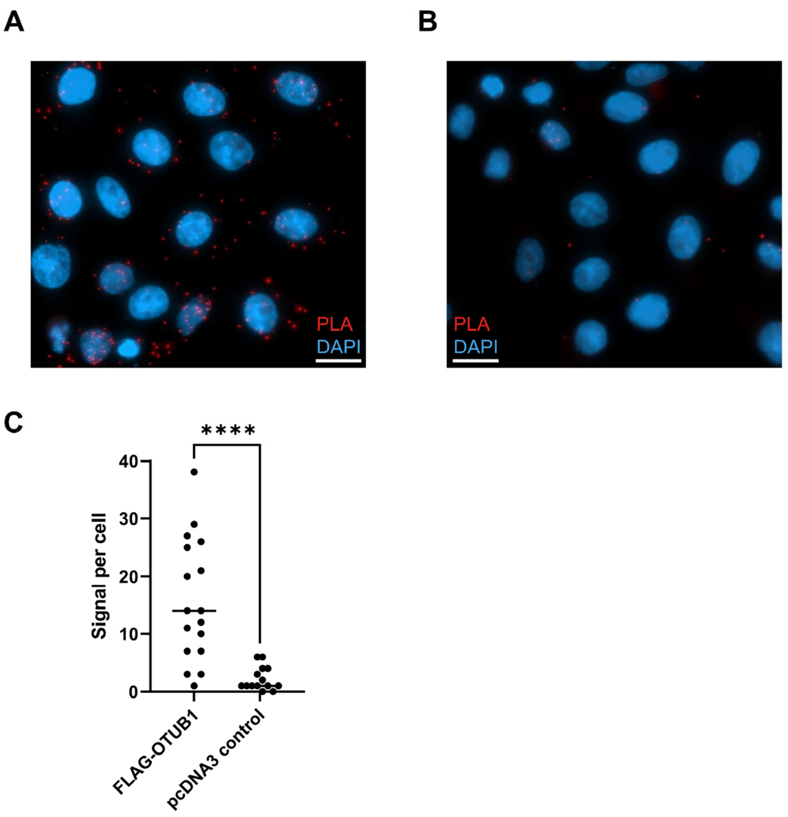

Figure S4. Proximity ligation assay indicates physical association between OTUB1 and c-MYC. (**A**) HeLa cells transfected with FLAG-OTUB1 were analyzed by proximity ligation assay using anti-MYC and anti-FLAG antibodies. Images were taken at 25× magnification and orthogonally projected following examination of 25 z-stacks (7.25 µm). Positive interaction between c-MYC and OTUB1 is indicated in red dots. DAPI was used as counterstain. The size bar indicates 20 µm. (**B**) Negative control proximity ligation using the same conditions as in (A) with HeLa cells transfected with the empty pcDNA3 control vector. (**C**) Analysis of proximity ligation signal shown in panels (**A**) and (**B**), which are representative fields of view ($p < 0.0001$ by Wilcoxon rank-sum test; bar indicates median).

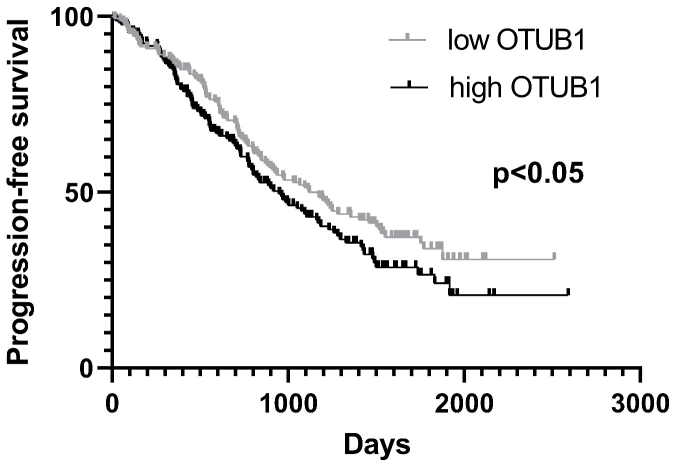

Figure S5. Multiple myeloma patients with elevated OTUB1 levels (top versus bottom quartile) showed worse clinical outcomes based on data from the CoMMpass study [1]. A Mantel-Cox analysis of the Kaplan-Meier plot indicates that OTUB1 shortens progression-free survival ($p < 0.05$).

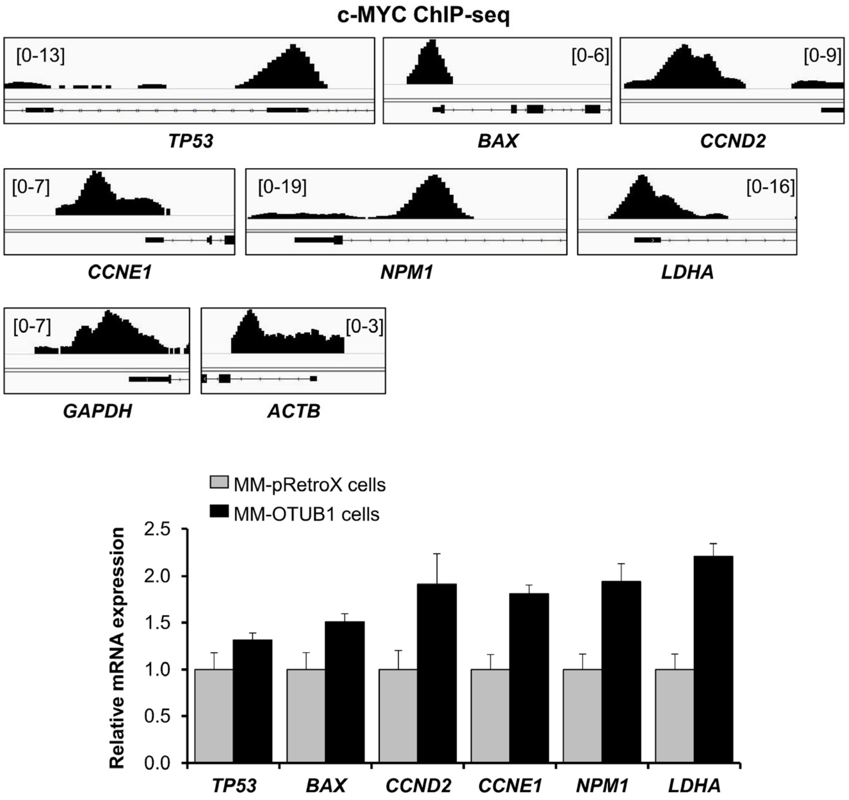

Figure S6. OTUB1 overexpression increases transcription of c-MYC target genes in MM.1S cells. Top panel: Gene tracks of c-MYC binding occupancy at the promoters of Tumor Protein P53 (TP53), BCL2 Associated X, Apoptosis Regulator (BAX), Cyclin D2 (CCND2), Cyclin E1 (CCNE1), Nucleophosmin 1 (NPM1), Lactate Dehydrogenase A (LDHA), Glyceraldehyde-3-Phosphate Dehydrogenase (GAPDH), and β-Actin (ACTB) promoters in MM.1S cells. The gene structure is shown at the bottom of each panel. The signal scale of the Integrative Genome Viewer (IGV) ChIP-seq tracks for a given gene are represented by the numbers in brackets. The genomic region on the x-axis spans 2 kb for all the regions. IGV snapshots were built using the publicly available ChIP-seq dataset deposited on the NCBI Gene Expression Omnibus repository (GEO accession number GSE36354; sample GSM894108). Due to the spurious binding of c-MYC to the ACTB promoter, this gene was used as reference for the subsequent RTqPCR assays. Bottom panel: OTUB1 overexpressing MM.1S cells showed elevated transcription of several c-MYC target genes ($p < 0.05$ based on the two-sided Student's *t*-test).

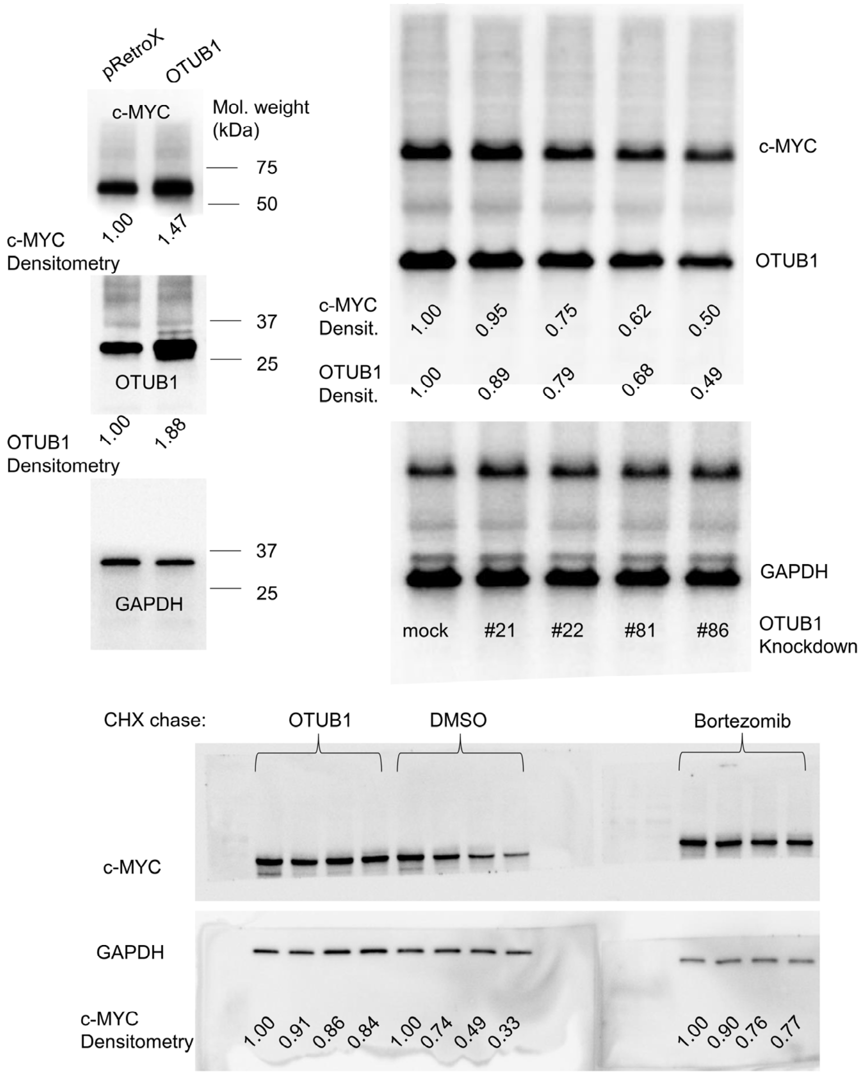

Figure S7. Uncropped Western blots shown in Figure 5. Densitometry readings were quantified with Image J version 1.52 and standardized to the respective GAPDH signal.

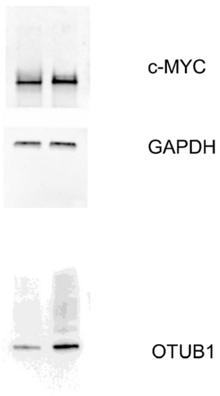

Figure S8. Uncropped Western blots shown in Figure 7.

Reference

1. Giusti, K. Company Profile: Multiple Myeloma Research Foundation. *Pers. Med.* **2012**, *9*, 333–336. https://doi.org/10.2217/pme.12.25.
